# Supplementary material for: A pathological phosphorylation pattern enhances tau cooperativity on microtubules and facilitates tau filament assembly
Source: Res Sq. 2025 Apr 10:rs.3.rs-6247226. Preprint. [Version 1] doi: 10.21203/rs.3.rs-6247226/v1 (PMC12036459; doi:10.21203/rs.3.rs-6247226/v1)
Supplement: 1 [file NIHPPRS6247226V1-supplement-1.pdf]

Supplementary Table 1: Site-specific phosphorylation of eukaryotic expressed 2N4R tau.

site	Degree of phosphorylation		site	Degree of phosphorylation	
	HEK tau	SF9 tau		HEK tau	SF9 tau
T17	0	(0.06 ± 0.08) %	T231	(97.47 ± 0.02) %	(99.81 ± 0.28) %
Y18	0	(0.01 ± 0.01) %	S235	(34.46 ± 37.78) %	(29.58 ± 40.68) %
Y29	0	(1.10 ± 1.55) %	S237	(25.96 ± 0.59) %	(49.17 ± 36.31) %
T30	(0.19 ± 0.27) %	(0.41 ± 0.58) %	S238	0	(0.03 ± 0.05) %
T39	0	(0.20 ± 0.28) %	S241	(17.30 ± 24.47) %	(4.80 ± 6.79) %
S46	(10.72 ± 15.16) %	(1.48 ± 1.93) %	T245	(0.02 ± 0.04) %	(0.01 ± 0.01) %
T50	(3.19 ± 4.51) %	0	S258	(16.84 ± 23.81) %	0
T52	0	(0.01 ± 0.02) %	S262	(0.50 ± 0.70) %	0
S56	0	(0.01 ± 0.02) %	T263	(0.16 ± 0.22) %	(0.07 ± 0.09) %
S61	0	(0.21 ± 0.30) %	S285	(0.06 ± 0.08) %	(0.003 ± 0.005) %
S68	(1.27 ± 1.64) %	0	S289	(0.01 ± 0.01) %	(0.01 ± 0.02) %
T69	(0.07 ± 0.10) %	0	S293	(0.72 ± 1.03) %	(2.31 ± 3.27) %
T71	(0.29 ± 0.21) %	0	S305	(0.84 ± 1.04) %	(0.56 ± 0.78) %
T76	0	(0.25 ± 0.35) %	Y310	(0.16 ± 0.04) %	(0.39 ± 0.52) %
T102	(5.70 ± 8.05) %	0	S316	(0.30 ± 0.43) %	0
T111	0	(0.01 ± 0.01) %	T319	(24.62 ± 34.81) %	0
S113	(0.14 ± 0.19) %	(0.02 ± 0.03) %	S320	(5.65 ± 8.00) %	0
T123	0	(0.02 ± 0.03) %	S324	(1.31 ± 0.17) %	(0.54 ± 0.77) %
T169	(4.55 ± 6.43) %	(1.08 ± 1.53) %	S341	(0.06 ± 0.09) %	0
T175	(3.58 ± 4.57) %	(7.78 ± 4.23) %	S352	(4.53 ± 6.41) %	(44.70 ± 63.22) %
T181	(72.42 ± 11.34) %	(66.42 ± 8.49) %	S356	(0.51 ± 0.58) %	(0.76 ± 0.76) %
S184	0	(0.37 ± 0.53) %	T361	(0.03 ± 0.04) %	(0.02 ± 0.02) %
S185	0	(0.18 ± 0.25) %	T373	(29.56 ± 41.80) %	0
S191	(0.60 ± 0.85) %	(10.80 ± 15.28) %	T377	(8.13 ± 11.50) %	0
S195	(0.97 ± 1.38) %	(2.54 ± 3.59) %	T386	0	(0.08 ± 0.11) %
Y197	(0.35 ± 0.50) %	0	Y394	(0.63 ± 0.89) %	(3.99 ± 5.64) %
S198	(0.25 ± 0.36) %	(22.99 ± 4.81) %	S396	(11.53 ± 15.69) %	(29.84 ± 32.75) %
S199	(3.45 ± 4.88) %	(4.82 ± 6.82) %	S400	(1.54 ± 0.13) %	(7.40 ± 9.85) %
S202	(16.81 ± 15.44) %	(47.06 ± 14.81) %	T403	(18.32 ± 25.69) %	(10.81 ± 15.29) %
T205	(1.42 ± 2.01) %	(0.99 ± 1.41) %	S404	(22.30 ± 25.04) %	(60.41 ± 17.79) %
S208	(0.07 ± 0. 10) %	(13.76 ± 19.46) %	S409	0	(0.12 ± 0.17) %
S210	(15.87 ± 22.14) %	(42.88 ± 56.19) %	T414	0	(0.12 ± 0.17) %
T212	(0.33 ± 0.47) %	(2.58 ± 2.95) %	S416	0	(1.44 ± 1.69) %
S214	0	(0.85 ± 0.87) %	S422	0	(31.71 ± 42.91) %
T217	(7.15 ± 10.08) %	(5.29 ± 1.17) %	T427	(10.61 ± 14.60) %	(0.83 ± 1.17) %
T220	(0.01 ± 0.01) %	(0.08 ± 0.12) %	S435	0	(0.02 ± 0.03) %

Average phosphorylation degree:

HEK tau: ~ 9.30 %   SF9 tau: ~ 10.58 %
